# Interventricular asynchrony in Chronic Thrombo Embolic Pulmonary Hypertension recovers after pulmonary endarterectomy: role of right ventricular wall stress

**DOI:** 10.1186/1532-429X-13-S1-P319

**Published:** 2011-02-02

**Authors:** J Tim Marcus, Gert Jan Mauritz, Sulaiman Surie, Joachim Bosboom, Jaap J Kloek, Anton Vonk-Noordegraaf

**Affiliations:** 1 VU University Medical Center, Amsterdam, Netherlands; 2 Academic Medical Center, Amsterdam, Netherlands

## Introduction

Interventricular mechanical asynchrony is a characteristic of right ventricular (RV) pressure overload. Myocardial wall stress may play a key role. Wall stress reduction by an intervention may provide evidence for this role.

## Purpose

To investigate the effect of right ventricular (RV) wall stress on Left-Right (L-R) asynchrony in patients with Chronic Thrombo-Embolic Pulmonary Hypertension (CTEPH).

## Methods

**Figure 1:**
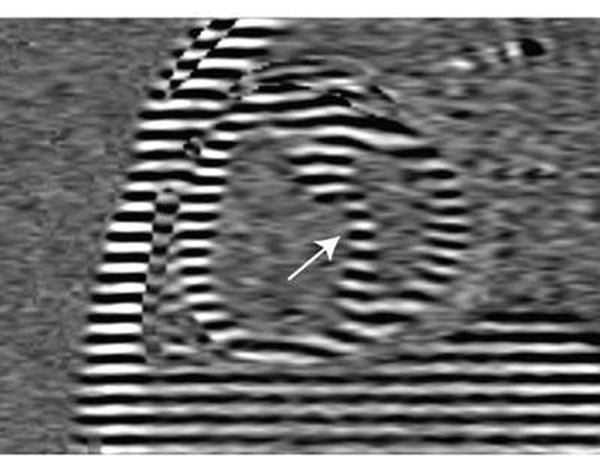
Short-axis tagged image at the time of peak RV shortening, acquired before pulmonary endarterectomy (PEA) in a CTEPH patient. Leftward ventricular septal bowing is denoted with the white arrow.

Reduction of RV wall stress was accomplished by pulmonary endarterectomy (PEA), which is the surgical removal of thrombo-emboli out of the pulmonary arteries. In 13 consecutive patients with CTEPH, MRI myocardial tagging (29 ms temporal resolution) was applied before and after PEA (fig 1). For the left ventricular (LV) free wall, septum (S) and RV free wall, the time to peak (T_peak_) of circumferential shortening was calculated.

Pulmonary Artery Pressure (PAP) was measured by right heart catheterization. For the RV free wall, the end-systolic wall stress was calculated by the Laplace law, before and after PEA.

## Results

Table 1 shows that after PEA, the synchrony in T_peak_ , RV peak strain and stroke volume have recovered.

**Table 1:** Strain parameters, stroke volume and RV wall stress before and after PEA

	Before PEA	After PEA	p
L-R delay in Tpeak (ms)	95 ± 61	2 ± 47	<0.001
S-R delay in Tpeak (ms)	136 ± 48	25 ± 20	<0.001
RV peak strain (%)	12 ± 3	17 ± 3	<0.05
Stroke volume (ml)	59 ± 13	72 ± 10	<0.001
RV wall stress (kPa)	39 ± 17	17 ± 10	<0.001

The reduction of L-R delay in T_peak_ was associated with the reduction in RV wall stress (r=0.67, p=0.004), but not with the reduction in systolic PAP.

## Discussion

The key role of RV wall stress has been proven in the present intervention study. Pulmonary hypertension leads to increased RV wall stress, which causes a prolongation of RV myocardial shortening. By consequence, the RV free wall continues shortening while the left ventricular (LV) wall is already in its early diastolic phase. Thereby the ventricular septum bows to the left. Thus the end of RV systole is very ineffective, and in addition the early LV filling is impaired. These effects both result in a loss of stroke volume.

## Conclusion

After PEA in CTEPH, the RV peak strain is resynchronized with the LV peak strain and regains its normal peak value. Reduction of RV wall stress plays a key role in this recovery.

